# A comparison of the characteristics of accidental substance-related acute toxicity deaths in Canada across life stages, 2016–2017

**DOI:** 10.24095/hpcdp.44.7/8.04

**Published:** 2024-07

**Authors:** Grace Yi-Shin Chang, Jingru Helen Ha, Jacqueline Burt, Fiona Kouyoumdjian, Katherine McKenzie, Shane Randell, Amanda VanSteelandt

**Affiliations:** 1 Substance-Related Harms Division, Health Promotion and Chronic Disease Prevention Branch, Public Health Agency of Canada, Ottawa, Ontario, Canada; 2 Office of Drug Research and Surveillance, Health Canada, Ottawa, Ontario, Canada; 3 Ontario Ministry of Health, Toronto, Ontario, Canada; 4 Lifespan Chronic Disease and Conditions Division, Health Promotion and Chronic Disease Prevention Branch, Public Health Agency of Canada, Ottawa, Ontario, Canada; 5 Centre for Emergency Preparedness, Public Health Agency of Canada, Ottawa, Ontario, Canada

**Keywords:** substance use, acute toxicity deaths, youth, adults, older adults, Canada

## Abstract

The acute toxicity (sometimes called “overdose” or “poisoning”) crisis has affected Canadians across all stages of life, including youth, adults and older adults. Our biological risks and exposures to substances change as we age. Based on a national chart review study of coroner and medical examiner data on acute toxicity deaths in 2016 and 2017, this analysis compares the burden of deaths and circumstances of death, locations of acute toxicity event and death, health history and substances contributing to death of people, by sex and life stage.

**Highlights**

- This analysis reveals key differences in the characteristics of acute toxicity deaths by sex and life stage, and suggests potential intervention points for each group.
- Many people across demographics were alone while using substances before the acute toxicity event, and many were alone when they died. Youth, particularly female youth, more often died in circumstances where someone might have been available to help by calling 911 or administering first aid and naloxone.
- For the people who were in contact with health care prior to their death, about one-quarter (24%–28%) of adults and older adults sought assistance for reasons related to pain. Youth more often sought assistance for a nonfatal acute toxicity event (13%–14%) or for mental health (particularly female youth, 21%) than people in other life stages.
- Multiple substances contributed to most deaths, and both pharmaceutical and nonpharmaceutical substances were common causes of death for all life stages and sexes. There are demographic differences in the specific substances contributing to death.

## Introduction

The acute toxicity (sometimes called “overdose” or “poisoning”) crisis has affected Canadians from all walks of life and of all ages—children, youth, adults including older adults have died. At the population level, our biological risks from substance use change as we age: our brains are not fully developed until our mid-20s;^1^ over time we can accumulate more diseases and disorders;^2^ and eventually our metabolism and ability to process substances slows down.^3^ Our exposures to substances also evolve with age: first exposures to nonmedical substance use are often in our youth;^4^ peer pressure to engage in nonmedical substance use changes over time;^4^ and we are more likely to have multiple prescriptions in later life.^5^

In this analysis, we compare characteristics of acute toxicity deaths across life stages for youth, adults and older adults. This analysis serves as an important baseline at the beginning of the acute toxicity crisis that can be used to measure change. It is intended to bridge previously published in-depth reports on youth^6^ and older adults,^7^ and compares broader life stages rather than the 5- or 10-year age groupings other reports use based on the same dataset.^8^,^9^

## Methods

This study was reviewed and approved by the Public Health Agency of Canada Research Ethics Board (REB 2018-027P), the University of Manitoba Health Research Ethics Board (HS22710) and the Newfoundland and Labrador Health Research Ethics Board (20200153).

For the purposes of this study, life stages are defined as youth (aged 12 to 24 years), adults (aged 25 to 59 years) and older adults (aged 60 plus years). Substances include alcohol, pharmaceutical and nonpharmaceutical drugs and chemicals not approved for human consumption (e.g. illegal drugs, nonpharmaceutical inhalants, industrial or household chemicals, or veterinary drugs). Based on a national retrospective chart review study of coroner and medical examiner data on all substance-related acute toxicity deaths from 1 January 2016 to 31 December 2017,^8^,^10^ we calculated the burden of accidental substance-related acute toxicity deaths and characteristics of people who died by sex and life stage. Table 1 lists the variables used in the analysis and their descriptions.

**Table 1:** Variables used to describe the burden of substance-related acute toxicity deaths and the characteristics of people who died, by sex and life stage, Canada, 2016–2017

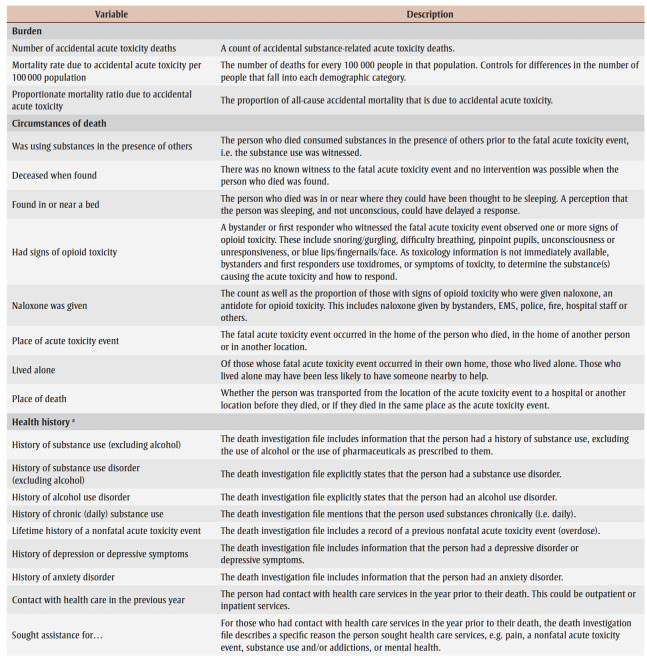 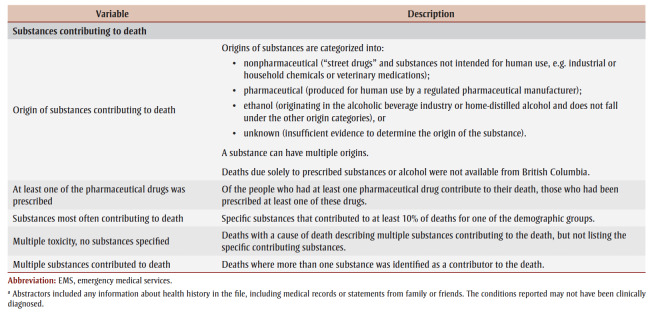

Burden is based on the number of deaths, mortality rate and proportionate mortality ratio due to accidental substance-related acute toxicity. Mortality rates were calculated with population counts from the 2016 Census^11^ as the denominator. To calculate the proportionate mortality ratios attributable to substance-related acute toxicity, we used data from Statistics Canada on all-cause accidental mortality counts by demographic group for the denominators. We included all-cause deaths with ICD-10 codes V01–V99 (transport accidents), W00–X59 (other external causes of accidental injury), Y85 (sequelae of transport accidents) and Y86 (sequelae of other accidents).

We also analyzed the circumstances of death, locations of the acute toxicity event and death, health history and substances contributing to death of people who died of accidental acute toxicity, by sex at birth and life stage, using the variables described in Table 1. We calculated the proportions of each group that had a given characteristic, and conducted Pearson chi-square tests to assess statistical differences across life stages and sex (*p* < 0.05). As information on the variables of interest are not always recorded in death investigation files, the results represent only the minimum proportions of people who had a given characteristic.

To protect privacy, all counts are randomly rounded to base 3 (i.e. values had different chances of being rounded to nearest multiples of 3) and counts less than 10 are suppressed.^10^ Since table totals were also independently rounded to base 3, the sum of values do not always equal the total. Proportions and mortality rates are calculated with rounded counts. 

## Results

Each of these demographic groups has been affected by the acute toxicity crisis in different ways. Acute toxicity accounted for 41% to 60% of all accidental deaths for youth and adults (Table 2). The mortality rate due to accidental acute toxicity was much higher for male adults (30 deaths per 100 000 population) than the other demographic groups (2.8–9.5 deaths per 100 000 population). Among the people who died of accidental acute toxicity, contacts with health care, circumstances of death and substances contributing to death varied by life stage and sex. 

**Table 2:** Burden, circumstances of death, documented health history and substances contributing to the deaths of people who died of accidental acute toxicity, by sex and life stage, Canada, 2016–2017

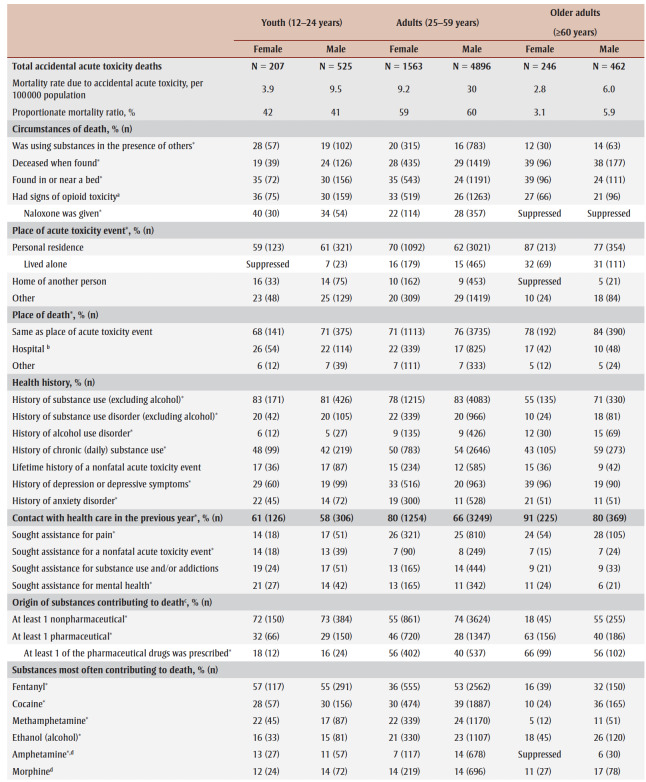 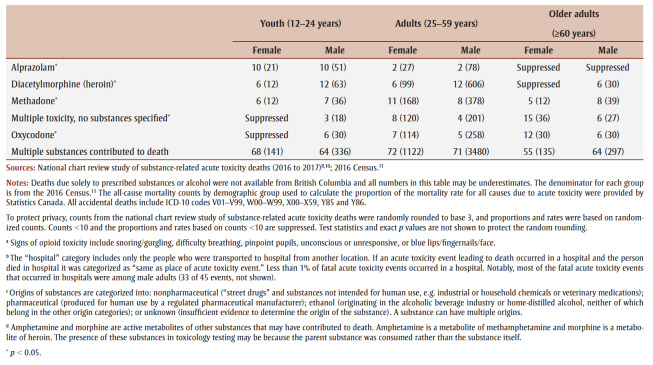

***Circumstances of accidental acute toxicity deaths***

- Older adults were less often using substances in the presence of others prior to their death (12%–14% vs. 16%–28%).
- Older adults were more often already dead when found compared with youth and adults (38%–39% vs. 19%–29%).
- Many people were found in or near a bed (24% to 39%) where their acute toxicity event could have been misinterpreted as sleep. Females were more often found in or near a bed.
- Among people who were reported to show signs of opioid toxicity before death, naloxone was less often administered to older adults (counts and proportions suppressed due to small numbers).
- For all life stages, the most frequent location for the acute toxicity event leading to death was the individual’s personal residence (59%–87%). Of those who had their acute toxicity event in their personal residence, older adults more commonly lived alone (31%–32% vs. 16% or less).
- Though less common across all life stages, youth and adults were more often at the home of another person compared to older adults (14%–16% and 9%–10%, respectively, vs. 5% or less).
- Most people died where the acute toxicity event happened (68%–84%). Female youth were most often transported to hospital before death (26%), and male older adults were least often transported to hospital (10%).

***Health history and previous contacts with the health system of people who died of accidental acute toxicity***

- Most people who died had a history of substance use (excluding alcohol). This was less common for female older adults (55%) than other demographic groups (71%–83%).
- Female older adults also had a history of substance use disorders (excluding alcohol) less frequently than other demographic groups (10% vs. 18%–22%).
- The frequency of alcohol use disorders increased with age (5%–6% among youth to 12%–15% among older adults).
- Male youth (42%) and female older adults (43%) had a history of chronic (daily) substance use less often than other demographic groups (48%–59%).
- Having a history of depression or depressive symptoms and of anxiety disorders was more frequent among females than males (29%–39% and 19%–22%, respectively, vs. 19%–22% and 11%–14%, respectively).
- Contact with health care services (inpatient or outpatient) in the year prior to death was more common with increased age (58%–61% for youth to 80%–91% for older adults).
- For the people who were in contact with health care prior to their death, there were no demographic differences in seeking care for substance use and/or addictions. About one-quarter (24%–28%) of adults and older adults sought assistance for reasons related to pain. Youth more often sought assistance for a nonfatal acute toxicity event (13%–14%) or for mental health (particularly female youth, 21%) than people in other life stages.

***Substances causing accidental acute toxicity deaths***

- A pharmaceutical substance contributed to death most often among female older adults (63% vs. 28%–46%).
- A nonpharmaceutical substance contributed to death most often among youth and male adults (72%–74%).
- Youth had a prescription for pharmaceutical drugs that contributed to their deaths less often than other groups (16%–18% vs. 40%–66%).
- Multiple substances contributed to most deaths (55%–72%).
- The most common substances contributing to death in youth were similar for both sexes (fentanyl, cocaine, methamphetamine, ethanol [alcohol] and amphetamine), but there were sex differences for the other age groups. For example, fentanyl contributed to a greater proportion of male adult deaths (53%) than female adult deaths (36%) and to male older adult deaths (32%) than female older adult deaths (16%).
- Fentanyl was a cause of more than half of fatal acute toxicity events of youth (55%–57%) and male adults (53%).

## Discussion

This analysis reveals key differences in the characteristics of acute toxicity deaths by sex and life stage and suggests potential intervention points for each group. Many people who died of acute toxicity had contact with health care in the year prior to their death. These encounters with the health care system provide earlier opportunities to identify and address the risk of a fatal acute toxicity event as well as unmet health and social needs that may contribute to substance use. About one in four adults and older adults were in contact with health care for reasons related to pain. Such contacts create an opportunity for discussions regarding pain management, including safe use of pain medications, seeking relief from other substances, and other available treatment options and services to help alleviate pain.

Youth, particularly female youth, more often died in circumstances where someone might have been available to help by calling 911 or administering first aid and naloxone (Table 2). It is important that potential witnesses to acute toxicity events be able to recognize and respond to the emergency, and have the right tools to help (e.g. a naloxone kit, a phone to call 911). Many people across demographics were alone while using substances before the acute toxicity event, and many were alone when they died. Removing the stigma of substance use is important so that those who are using substances alone can find greater safety with others. Supporting connections to laypeople trained in overdose prevention or formalized supervised consumption services could help prevent these deaths.

Multiple substances contributed to most deaths, and both pharmaceutical and nonpharmaceutical substances were common causes of death for all life stages and sexes (Table 2). When a pharmaceutical substance contributed to death, many people, and particularly older adults and female adults, had been prescribed the substance that caused their death. The involvement of multiple substances in an acute toxicity event is the norm, and the potential combined harms of substances are an important consideration for prescribing practices (e.g. management of multiple prescriptions), patient education, harm reduction programs, drug checking services and drug alerts. 

In this study, we were unable to differentiate between whether the multiple substances involved were intentionally or unintentionally consumed. Initiatives to address the toxic drug supply would benefit all demographics, as would a harm reduction approach to prescribing that emphasizes patient education about the risks of their prescription drugs, their risks in combination with other substances and the risks of diversion.^12^,^13^

***Strengths and limitations***

A chart review of death investigation data allowed for more detailed analysis of patterns in substance-related acute toxicity deaths for different demographic groups. This is particularly true for the circumstances surrounding the death, as there is limited contextual information captured in other reporting systems.

Death investigation protocols vary across the country, and information about these variables is not always consistently available in death investigation files. Age, sex and manner of death were complete for all records, but for other characteristics, these are the minimum proportions of people who died of substance-related acute toxicity that had a given characteristic, and may underestimate the true number.

## Conclusion

Acute toxicity is a major cause of accidental deaths among youth and adults in Canada, and entirely preventable. Contextual information from coroner and medical examiner files, even where some of the information we seek is missing, reveals patterns and potential opportunities to prevent further acute toxicity deaths for specific demographic groups, including through focused interventions across the life course. These patterns may have changed since the study period of 2016 to 2017, particularly during the COVID-19 pandemic, but these results serve as an important baseline to measure the impacts of interventions implemented in the intervening years. 

## Acknowledgements

We would like to acknowledge our collaborators at the offices of chief coroners and chief medical examiners across Canada for providing access to their death investigation files. We would also like to thank Brandi Abele, Matthew Bowes, Songul Bozat-Emre, Jessica Halverson, Dirk Huyer, Beth Jackson, Graham Jones, Jennifer Leason, Regan Murray, Erin Rees, Jenny Rotondo and Emily Schleihauf for their contributions in the development of the national chart review study on substance-related acute toxicity deaths. 

## Funding

This study was funded by the Public Health Agency of Canada. 

## Conflicts of interest

The authors report no conflicts of interest.

## Authors’ contributions and statement

GC: Conceptualization, data curation, formal analysis, writing – original draft, writing – review & editing. 

JH: Conceptualization, data curation, formal analysis, writing – original draft, writing – review & editing.

JB: Conceptualization, data curation, writing – original draft, writing – review & editing.

FK: Conceptualization, writing – original draft, writing – review & editing.

KM: Conceptualization, writing – original draft, writing – review & editing.

SR: Conceptualization, writing – original draft, writing – review & editing.

AV: Conceptualization, data curation, formal analysis, project administration, supervision, writing – original draft, writing– review & editing. 

The content and views expressed in this article are those of the authors and do not necessarily reflect those of the Government of Canada, the data providers or the funders.
